# Supplementary material for: Inhibition of DNA Repair by Inappropriate Activation of ATM, PARP, and DNA-PK with the Drug Agonist AsiDNA
Source: Cells. 2022 Jul 8;11(14):2149. doi: 10.3390/cells11142149 (PMC9320633; doi:10.3390/cells11142149)
Supplement: Supplementary file 1 [file cells-11-02149-s001.zip › cells-1706795-supplementary.pdf]

Supplementary Materials and Methods

Quantification of Cy5.5-AsiDNA uptake by Flow cytometry

AsiDNA conjugated to cyanine 5.5 (Cy5.5®) (Eurogentec S.A., Belgium) at 150 µg/ml was incubated with tumor cells for 1 h up to 120 h in complete medium. At each time points, the cells were trypsinized, and resuspended in 1X PBS. The cell suspensions were analysed by flow cytometry. The fluorescence intensity of each sample was recorded on a FACS LSR Fortessa™ X-20 (BD Biosciences), and the data were analyzed using FlowJo software (Tree Star).

ELISA Assay for PARylation

A sandwich ELISA was used to detect Poly(ADP-Ribose) (PAR) polymers. Cells were boiled in PathScan Sandwich ELISA Lysis Buffer (Cell Signaling Technology, #7018) supplemented with 1mM PMSF (Phenylmethanesulfonyl Fluoride, Sigma). The protein levels were determined using the BCA protein assay kit (Thermo Scientific, #23250). Each cell extract was diluted in three different concentrations in Superblock buffer (Thermo Scientific, #37515) prior to the ELISA Assay. A 96-well white opaque polystyrene plate (Thermo Scientific, Pierce) was coated with 100 µl per well carbonate buffer (1.5g/l sodium carbonate Na_2CO_3, 3g/l $NaHCO_3$) containing the capture antibody (mouse antiPAR at 4µg/ml, Trevigen #4335) overnight at 4°C, washed 8 times with PBST solution (PBS 1x/ 0.1% Tween) and saturated with Superblock buffer at 37°C for 1h. Then, diluted cell extract were applied to each well in duplicate and incubated overnight at 4°C, after which it was washed 8 times with PBST solution. The detection antibody (Rabbit anti-PAR, Trevigen #4336, diluted 1/1000 in PBS/2% milk/1% mouse serum) was added and incubated for 1h at room temperature. After washing 8 times in PBST, secondary antibody HRP-conjugated anti-rabbit (Abcam #ab97085, diluted 1/5000 in PBS/2% milk/1% mouse serum) was added to each well for 1h and washed 8 times with PBST solution.

To readout, 75µl of substrate for the enzyme (Supersignal Pico, Pierce, #37070) was added to each well. The optical absorbance (OD 425 nm) of samples was mesured and compared to a serial dilution of the pure PAR (Enzo, #ALX-202-043) to determine the amount of PAR polymers.

Western blot

For immunoblotting of proteins with molecular weight <100 kDa, cells were lysed by scraping into Laemmli buffer supplemented by protease and phosphatase inhibitors and boiling for 10 min. For proteins above 100 kDa, the trichloroacetic acid precipitation method (TCA 15%) was used before a lysis in Tris-HCl 100mM, EDTA 10mM, SDS 1% supplemented by protease and phosphatase inhibitors. The protein levels were normalized with the BCA protein assay kit (Thermo Scientific, #23250). Proteins were separated by SDS-PAGE in 12% or 5% polyacrylamide gels, transferred to nitrocellulose membranes, blocked with Odyssey buffer (LI-COR Biosciences, # 927-60001) for 1 h and hybridized overnight at 4°C with primary antibody diluted in Odyssey buffer. Blots were incubated with goat IRdye secondary antibodies at 1/10 000 dilution (LI-COR Biosciences, IRdye 680 or 800) imaged and quantified with the Odyssey Infrared Imaging System (LI-COR Biosciences).

Following primary antibodies were used: rabbit Phospho_ATM polyclonal at 1/100 (Abcam #ab2888), rabbit Phospho-HSP90α (Thr5/7) at 1/1000 (Cell Signaling Technology #3488), mouse HSP90 alpha polyclonal at 1/1000 (Abcam #Ab13492), mouse γ-H2AX (Ser139) at 1/2000 (Millipore #05-636), rabbit P-DNA-PKcs (S2056) at 1µg/ml (Abcam #ab18192), mouse P-DNA-PKcs (Thr2609) at 1/500 (Abcam #ab18356), mouse anti-DNA-PKcs Ab-4 cocktail at 1/100 (Thermo Scientific, #MS-423-P).

Supplementary figures

A)

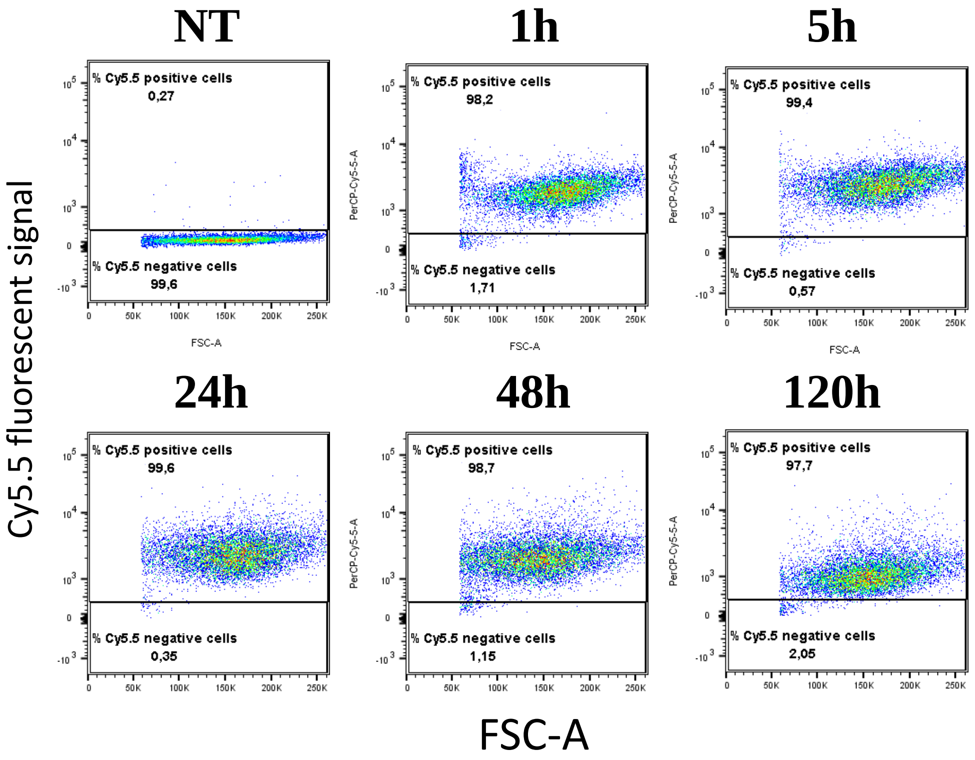

B)

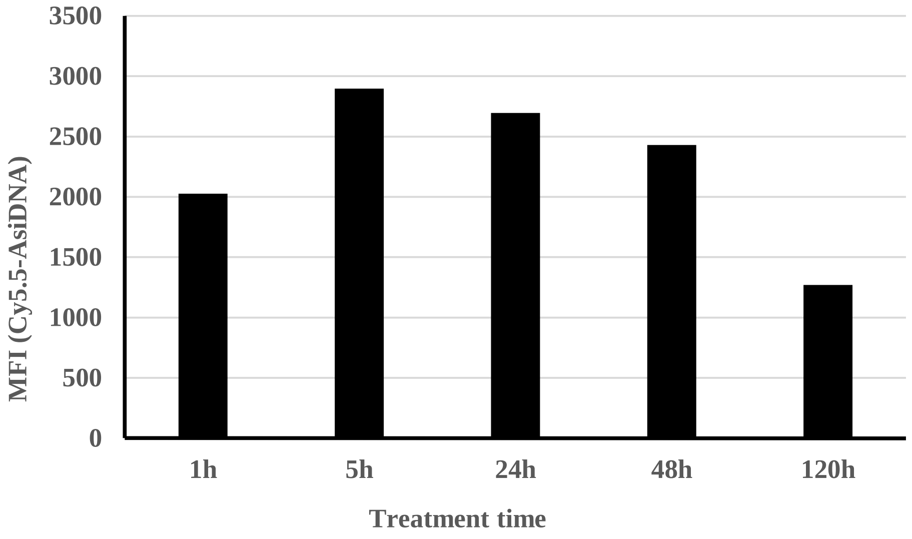

Figure S1. Uptake of AsiDNA in SK28 tumor cells as a function of the incubation time. (A) Flow-cytometry bivariate dot plot of SK28 cells emitting fluorescence due to the uptake of Cy5.5-AsiDNA. (B) Mean fluorescence intensity (MFI) of Cy5.5-AsiDNA taken up by the cells as a function of the treatment time.

Figure S1

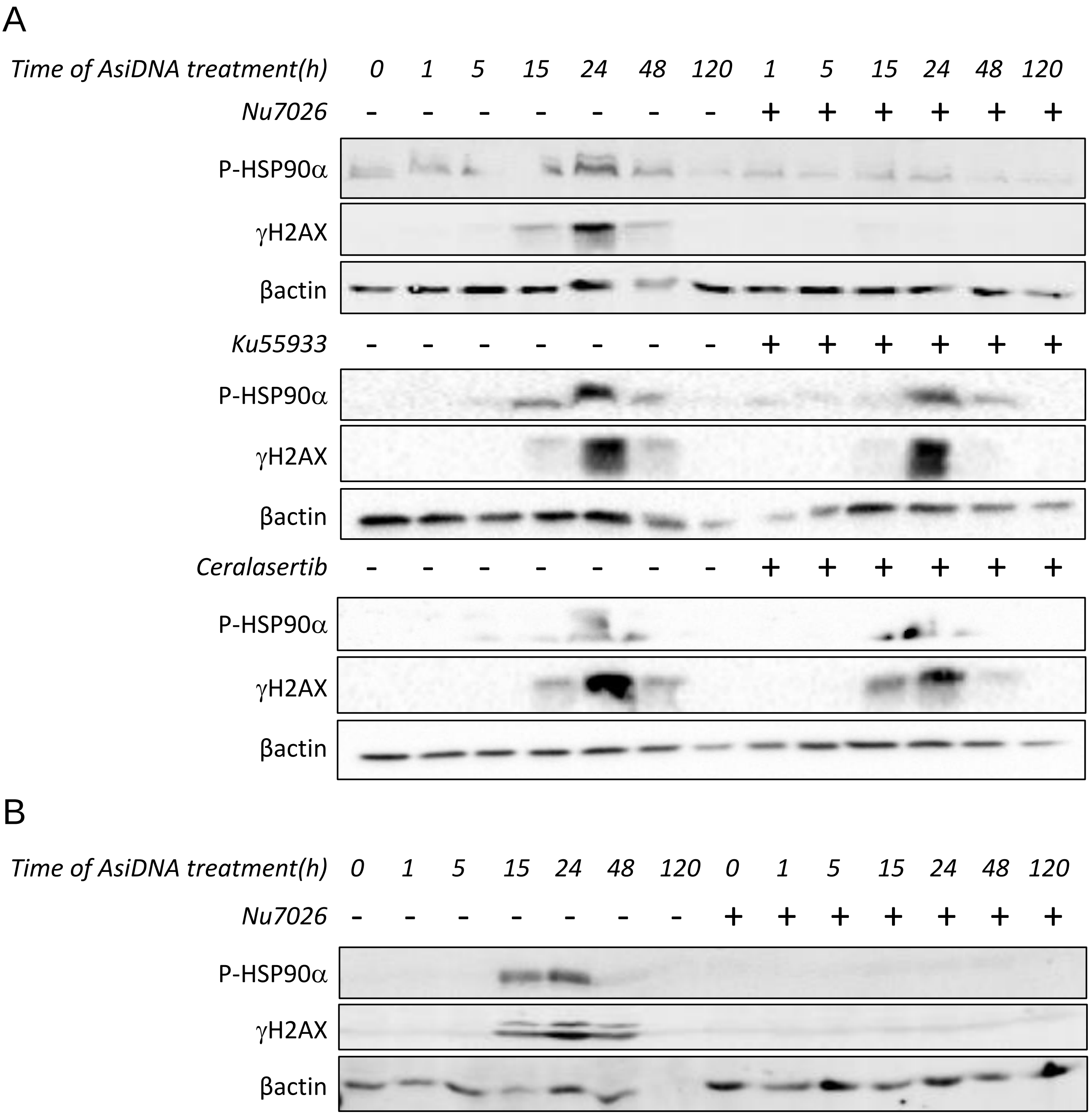

Figure S2 : Phosphorylation of HSP90 and H2AX is DNA-PK dependent upon AsiDNA treatment. The kinetics of H2AX and HSP90 phosphorylation in SK28 (**A**) and MRC5sv (**B**) pre-treated or not with DNA-PK inhibitor (NU7026), ATM inhibitor (Ku55933) or ATR inhibitor (ceralasertib), and further exposed to AsiDNA during different times were analyzed by western blot.

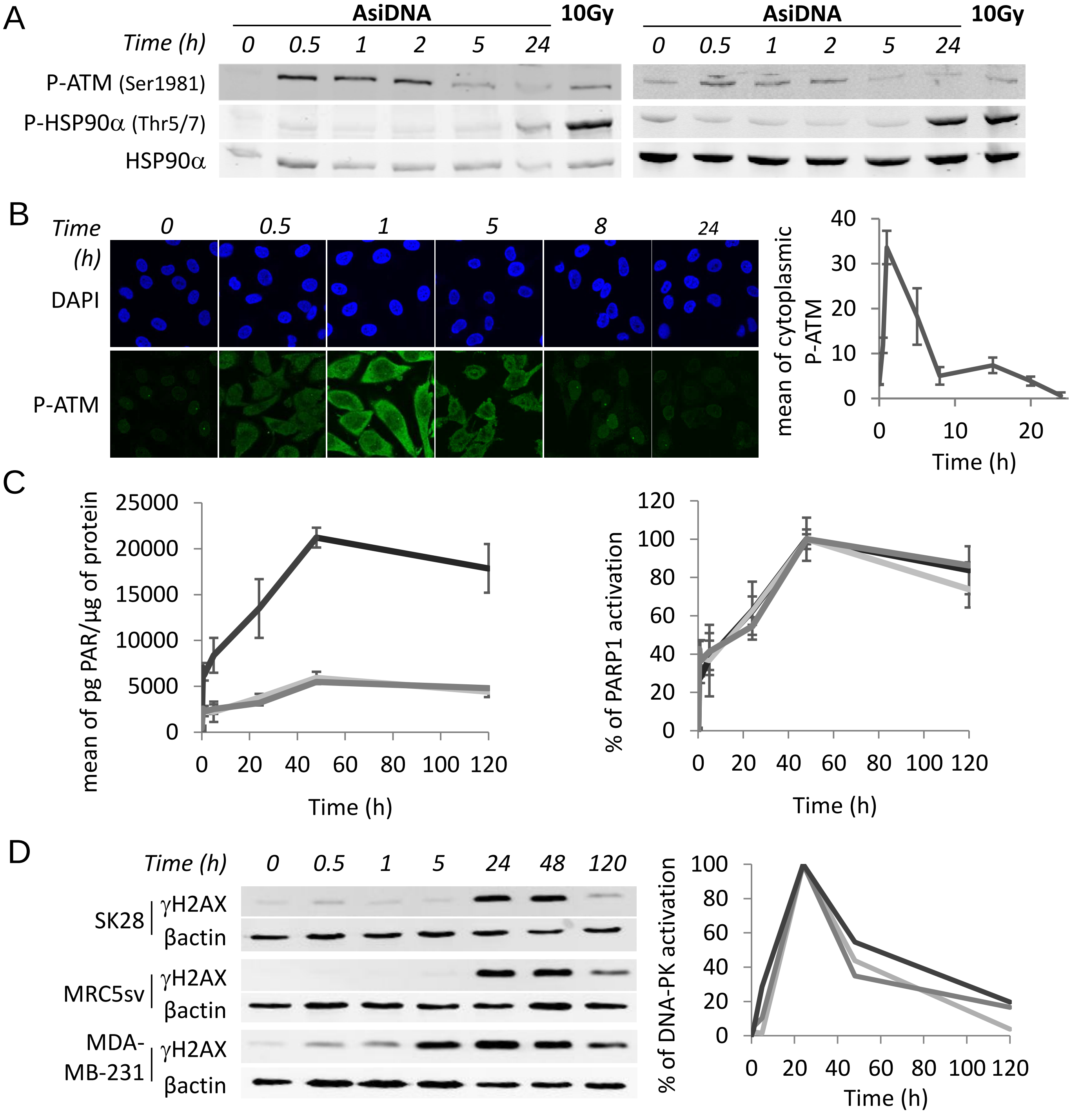

Figure S3 : Kinetics of activation of ATM, PARP1 and DNA-PK during AsiDNA treatment. (A) Kinetics of ATM autophosphorylation and phosphorylation of HSP90α in MRC-5sv treated by AsiDNA during different times was analyzed by western blot (B) Kinetic of ATM autophosphorylation in cytoplasm was analyzed by microscopy after immuno-fluorescent labeling. Quantification is indicated in right panel (C) Kinetic of PARylation by PARP1 in SK28 (dark gray), MRC-5sv (light gray) and MDA-MB-231 (gray) cell line treated by AsiDNA, measured by ELISA sandwich. Mean of 2 to 5 replicates per time point. Left panel: total amount of PAR per µg of protein; Right panel: relative value to maximum of PAR concentration (D) Kinetics of H2AX phophorylation at serine 139 in SK28, MRC-5sv and MDA-MB-231 cell line treated by AsiDNA. Left panel: exemple of western analysis ; Right panel: Quantification of the relative value of γH2AX/βactin in SK28 (dark gray), MRC-5sv (light gray) and MDA-MB-231 (gray) cell line

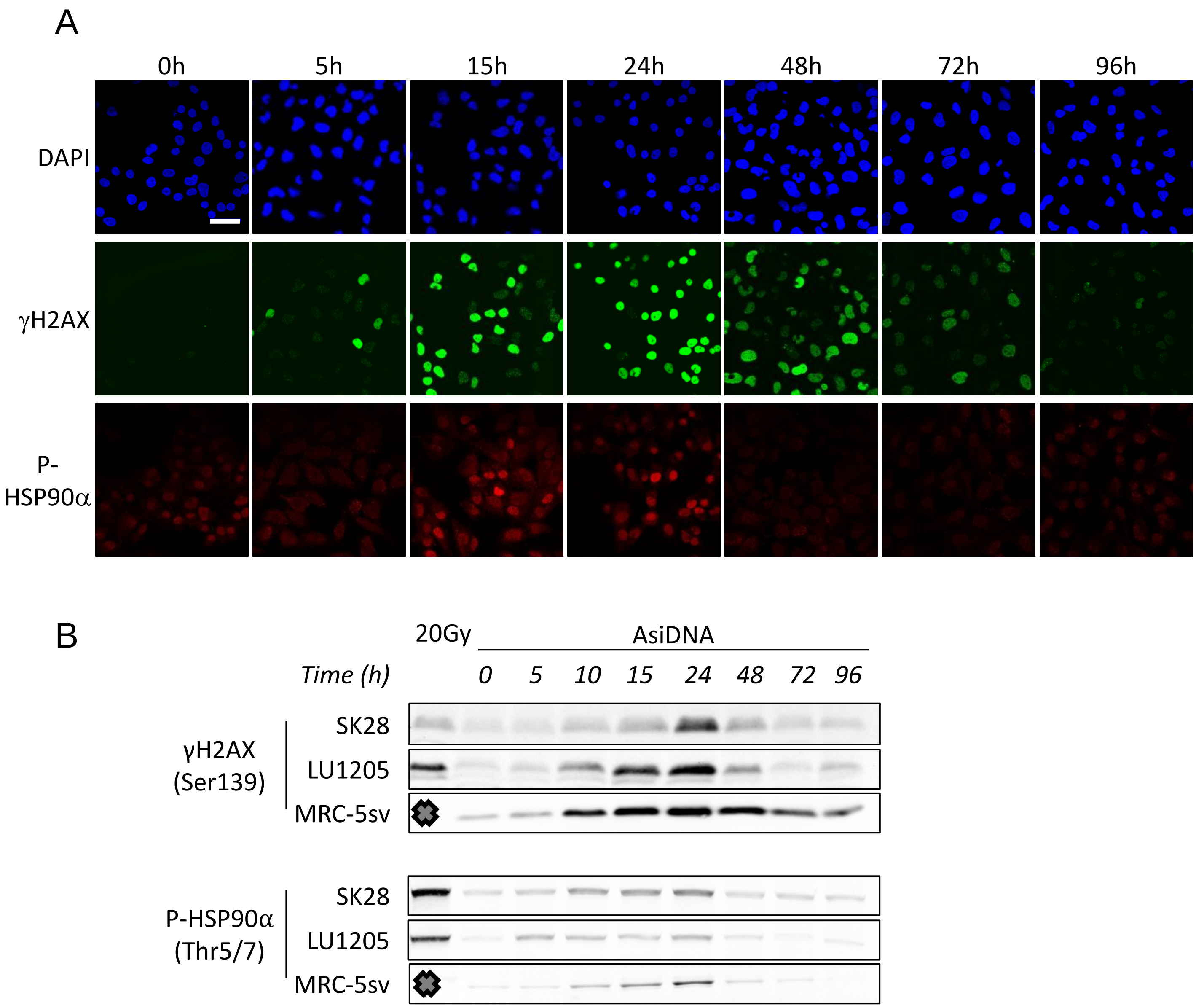

Figure S4 : Kinetics of phosphorylation of P-HSP90α and γH2AX. (A) Immunostaining in AsiDNA treated MRC-5sv. Scale bar: 50 µm. (B) Western Blot detection of γH2AX and P-HSP90α in SK28, Lu1205 and MRC-5sv cell lines. Cross: no extract.

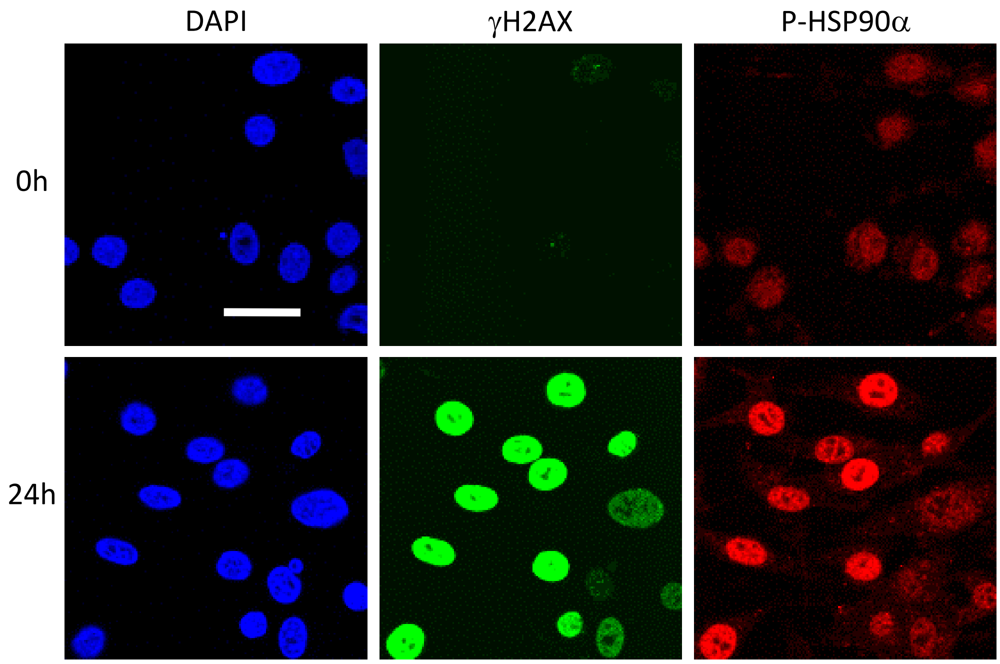

Figure S5 : Nuclear localisation of P-HSP90α and γH2AX. Immunostaining of P-HSP90α and γH2AX in SK28 cell line after 24h of AsiDNA treatment. Scale bar : 30 µm

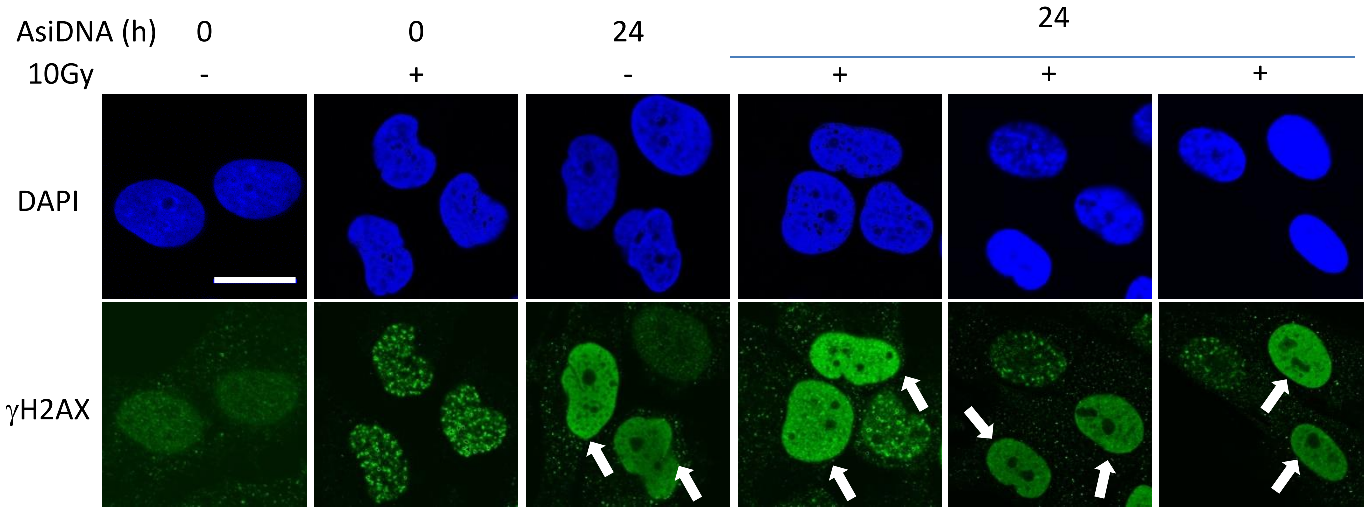

Figure S6 : Phosphorylation of H2AX. Immunostaining of γH2AX in MRC-5sv cell line treated or not with AsiDNA and exposed or not to 10Gy irradiation. White arrows: cells with pan-nuclear γH2AX. Scale bar : 20 µm

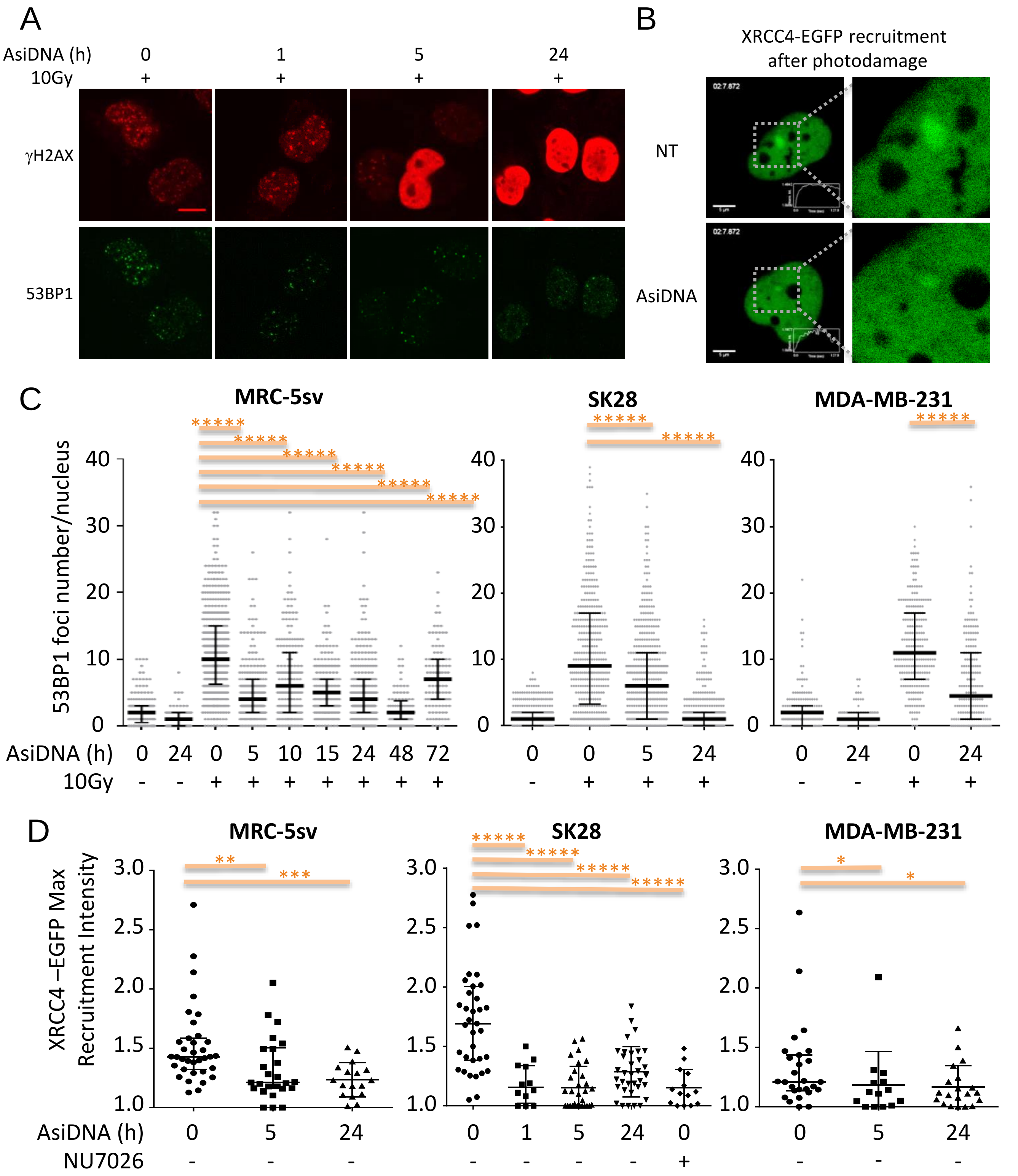

Figure S7 : Inhibition by AsiDNA of 53BP1 and XRCC4 recruitment on DNA damage. (A) Inhibition of 53BP1 recruitment after irradiation at 10Gy in MRC-5sv. Scale bar : 10µm (B) Inhibition of XRCC4-EGFP recruitment after laser irradiation in MRC-5sv. Scale bar : 5µm (C) Quantification of 53BP1 foci per nucleus in different cell lines pretreated for different times with AsiDNA before irradiation at 10Gy (D) Quantification of XRCC4-EGFP recruitment after photo-DNA-damage induction by laser 800nm in cells pretreated for different times with AsiDNA, values are corrected with photobleaching and background. Mann-Whitney test : ***** p value <0.00001, *** p value <0.001, **p value <0.01, *p value <0.04.

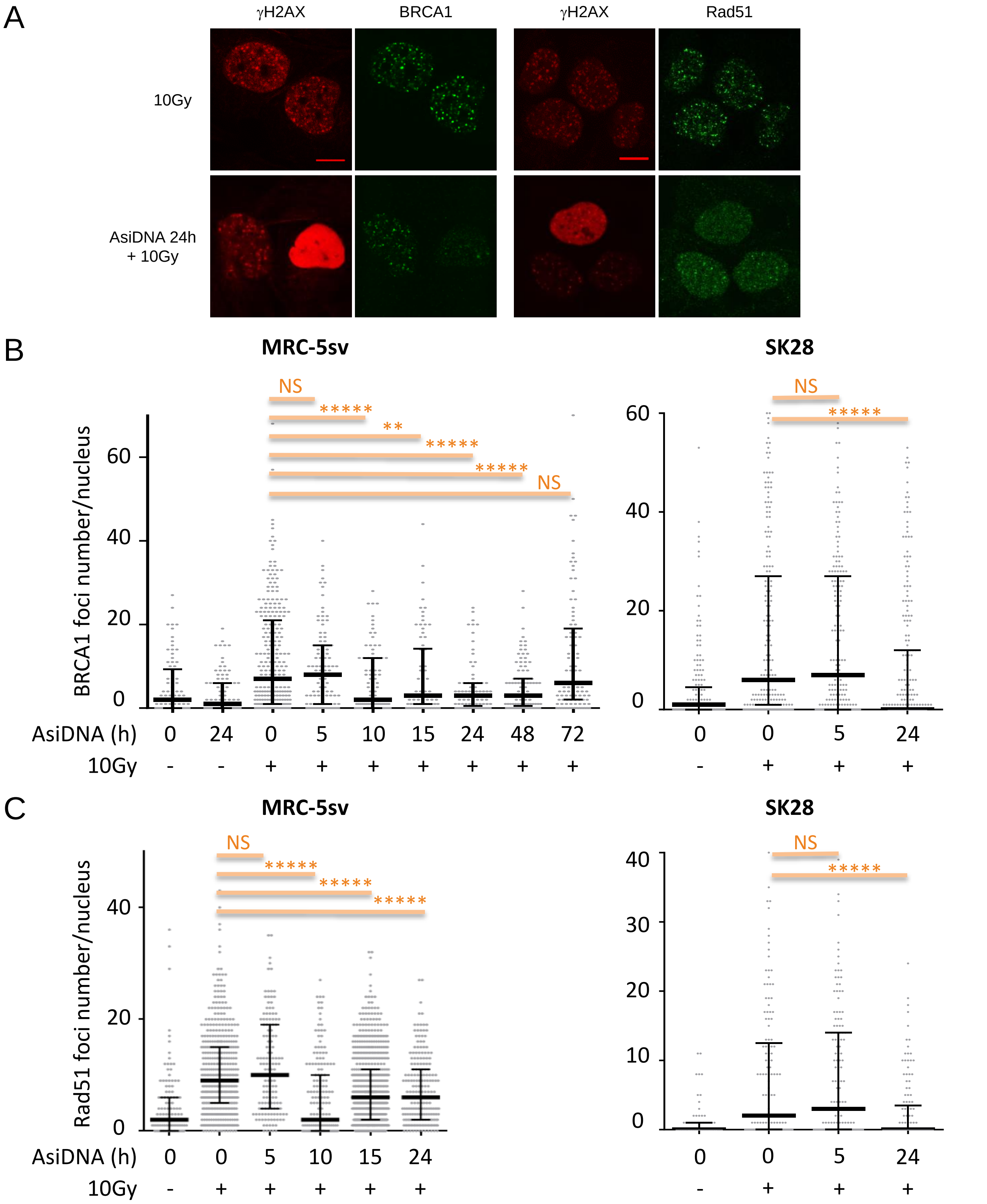

Figure S8 : HR Inhibition. (A) Inhibition of BRCA1 and Rad51 recruitment after irradiation in MRC-5sv cells pretreated 24h with AsiDNA and exposed to 10Gy. Scale bar : 10 µm (B) Quantification of BRCA1 foci per nucleus on immunofluoresence images after AsiDNA pretreatment and 10Gy irradiation (C) Quantification of Rad51 foci per nucleus. Mann-Whitney test : ***** p value <0.00001, **p value <0.01, NS : not significant.

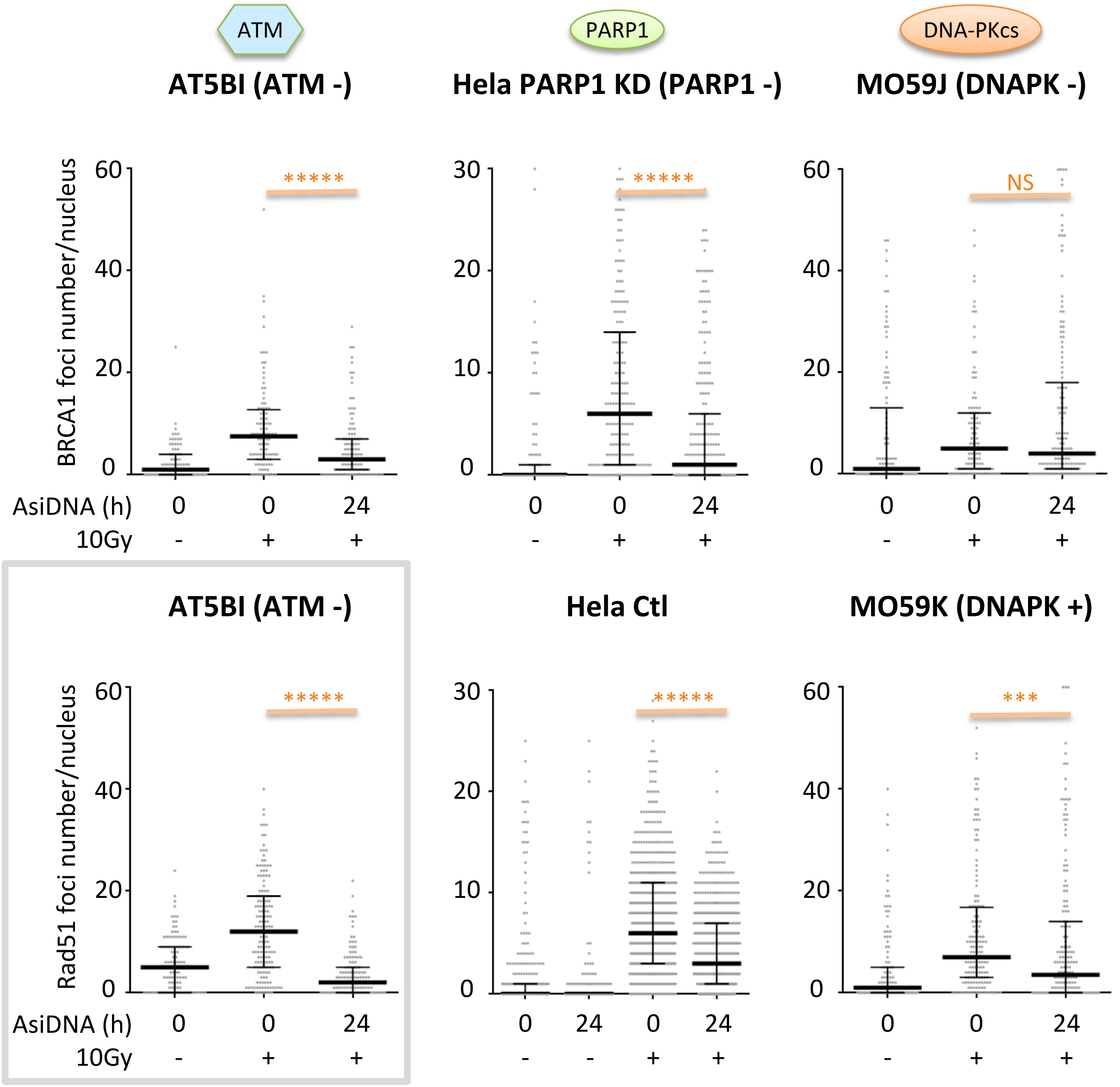

Figure S9 : BRCA1 inhibition of recruitment at DNA damage depend on DNA-PK after 24h of AsiDNA treatment. Quantification of BRCA1 or Rad51 foci per nucleus on immunofluoresence images after AsiDNA treatment +/- an irradiation of 10Gy in ATM, PARP1 or DNA-PK deficient cells. Mann-Whitney test : ***** p value <0.00001, *** p value <0.001, NS : not significant.

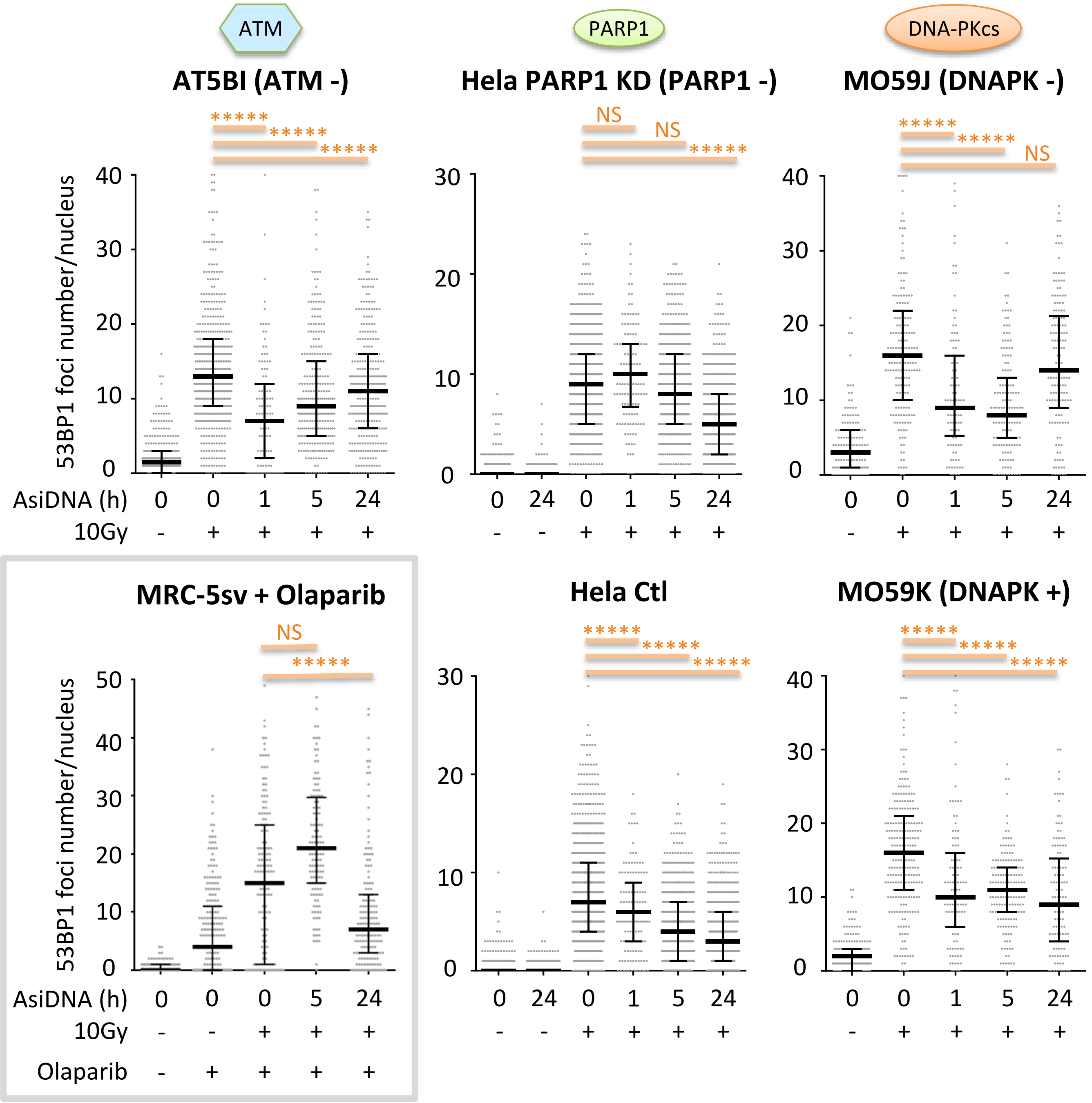

Figure S10 : Inhibition of 53BP1 recruitment at DNA damage site depends on PARP1 at early time points and DNA-PK after 24h of AsiDNA treatment. Quantification of 53BP1 foci per nucleus on immunofluoresence images after AsiDNA treatment +/- 10Gy irradiation in ATM, PARP1 or DNA-PK deficient cells or MRC-5sv pretreated by PARP1 inhibitor Olaparib. Mann-Whitney test : ***** p value <0.00001, NS : not significant.

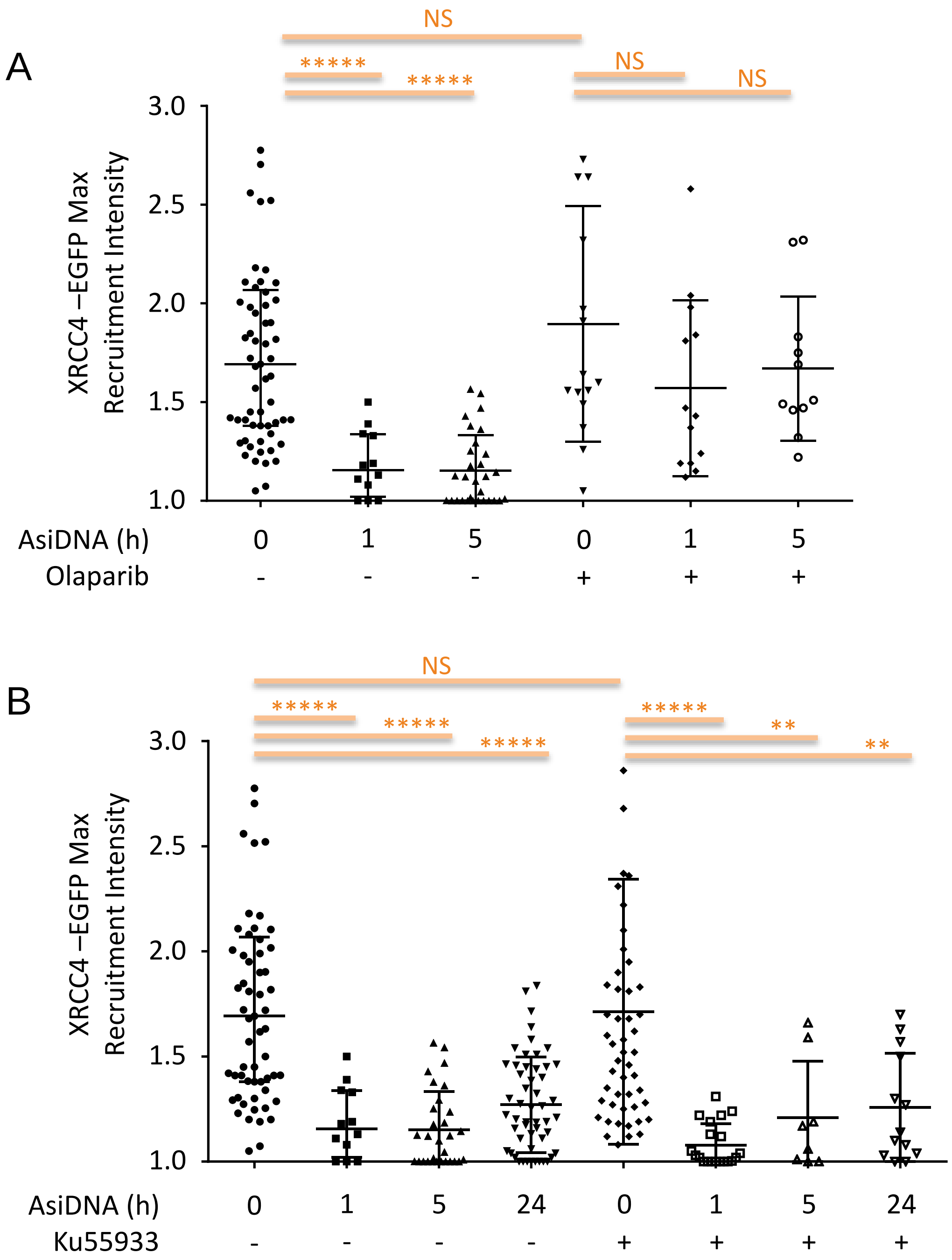

Figure S11 : Inhibition of XRCC4 recruitment at DNA damage site depends on PARP1 at early time points and is independent of ATM at all timepoints after AsiDNA treatment. Quantification of XRCC4-EGFP recruitment after photo-DNA-damage induction by laser 800nm in SK28 cells pretreated or not with ATM or PARP inhibitors and treated for different times with AsiDNA, values are corrected with photobleaching and background. Mann-Whitney test : ***** p value <0.00001, **p value <0.01, NS : not significant.
